# Design and Characterisation of a Randomized Food Intervention That Mimics Exposure to a Typical UK Diet to Provide Urine Samples for Identification and Validation of Metabolite Biomarkers of Food Intake

**DOI:** 10.3389/fnut.2020.561010

**Published:** 2020-10-21

**Authors:** Naomi D. Willis, Amanda J. Lloyd, Long Xie, Martina Stiegler, Kathleen Tailliart, Isabel Garcia-Perez, Edward S. Chambers, Manfred Beckmann, John Draper, John C. Mathers

**Affiliations:** ^1^Human Nutrition Research Centre, Population Health Sciences Institute, Newcastle University, Newcastle-upon-Tyne, United Kingdom; ^2^Institute of Biological, Environmental and Rural Sciences, Aberystwyth University, Aberystwyth, United Kingdom; ^3^Nutrition and Dietetic Research Group, Division of Diabetes, Endocrinology and Metabolism, Department of Medicine, Imperial College London, London, United Kingdom

**Keywords:** dietary intake, metabolomics, free-living participants, biomarkers, radomized control trial

## Abstract

Poor dietary choices are major risk factors for obesity and non-communicable diseases, which places an increasing burden on healthcare systems worldwide. To monitor the effectiveness of healthy eating guidelines and strategies, there is a need for objective measures of dietary intake in community settings. Metabolites derived from specific foods present in urine samples can provide objective biomarkers of food intake (BFIs). Whilst the majority of biomarker discovery/validation studies have investigated potential biomarkers for single foods only, this study considered the whole diet by using menus that delivered a wide range of foods in meals that emulated conventional UK eating patterns. Fifty-one healthy participants (range 19–77 years; 57% female) followed a uniquely designed, randomized controlled dietary intervention, and provided spot urine samples suitable for discovery of BFIs within a real-world context. Free-living participants prepared and consumed all foods and drinks in their own homes and were asked to follow the protocols for meal consumption and home urine sample collection. This study also assessed the robustness, and impact on data quality, of a minimally invasive urine collection protocol. Overall the study design was well-accepted by participants and concluded successfully without any drop outs. Compliance for urine collection, adherence to menu plans, and observance of recommended meal timings, was shown to be very high. Metabolome analysis using mass spectrometry coupled with data mining demonstrated that the study protocol was well-suited for BFI discovery and validation. Novel, putative biomarkers for an extended range of foods were identified including legumes, curry, strongly-heated products, and artificially sweetened, low calorie beverages. In conclusion, aspects of this study design would help to overcome several current challenges in the development of BFI technology. One specific attribute was the examination of BFI generalizability across related food groups and across different preparations and cooking methods of foods. Furthermore, the collection of urine samples at multiple time points helped to determine which spot sample was optimal for identification and validation of BFIs in free-living individuals. A further valuable design feature centered on the comprehensiveness of the menu design which allowed the testing of biomarker specificity within a biobank of urine samples.

## Introduction

The amount and pattern of foods and beverages consumed influence gene expression (1) and are major determinants of multiple health outcomes (2). Despite this centrality in the atiology of health and disease, the estimation of habitual dietary intake remains difficult (3). Conventional tools based on dietary self-report are tedious and time-consuming for both study participants and researchers. Dietary misreporting is common and substantial (4) and is exacerbated in those who are overweight or obese (5). Whilst the development of digital tools to assist with dietary recording may reduce the workload for respondents and researchers (6), use of such tools does not eliminate the subjectivity and biases inherent in approaches based on self-report. To improve measurements of dietary intake, there is a need to develop strategies for the objective identification, validation and deployment of suitable biomarkers (7).

Biomarkers of food intake (BFIs) assessed in body fluids or easily-accessible tissues offer potential alternative, objective routes to estimating dietary exposure (8). Measurement of BFIs (9, 10) could overcome some of the limitations of traditional dietary assessment methodologies by providing additional objective estimates of food exposure (11). Such biomarkers are of two main types: (i) those biomarkers which attempt to estimate a major class of nutrients e.g., urinary nitrogen excretion as an index of dietary protein consumption (12) and (ii) biomarkers which attempt to estimate intake of specific foods or food constituents (13). Most foods contain large numbers of characteristic metabolites many of which are cataloged in comprehensive databases of food composition, e.g., FoodB (developed by the University of Alberta, Canada: www.foodb.ca) and of selected food components, e.g., Phenol Explorer (developed by INRA: www.phenol-explorer.eu). However, when foods are consumed, they undergo metabolic transformation during digestion and absorption by enterocytes and colonocytes, by bacteria within the gut lumen and by Phase 1 and Phase 2 enzymes within the liver. Therefore, although the patterns of metabolites present in blood or urine reflect what has been consumed, the specific metabolites in such body fluids may differ substantially from those ingested (14).

We, and others, have applied metabolomics approaches to blood, saliva and urine in human studies to discover novel BFIs (8, 15–22). We have focused on urine as the body fluid of choice because of the ease of collection and the fact that, in contrast with blood, it provides an integrated estimate of exposure over several hours and because of technical advantages in sample preparation for metabolomics assay (8). This has led to the identification of a substantial number of putative biomarkers of individual foods (23) and to potential biomarkers of the overall healthfulness of the diet (24). Consensus guidelines for the critical assessment of candidate BFIs have been proposed recently (10). Although valuable, these guidelines focused on qualifying the utility of individual BFIs to monitor exposure to specific foods/food groups. Food intervention studies to determine the impact of individual dietary components on health form a large component of nutrition research but equally important is the need for approaches to assess overall dietary exposure in epidemiological studies and clinical trials (24). Whilst these discoveries and developments are encouraging, several challenges remain, associated particularly with the deployment of BFI technology in real world settings (Table 1).

**Table 1:** Challenges associated with the design of a food intervention study to develop and assess deployment of BFI technology to monitor overall dietary exposure.

(1) Providing opportunity to expand the discovery of biomarkers to include as many commonly-consumed foods as possible
(2) Ensuring structured exposure to a sufficiently comprehensive range of foods to mimic diets typical of a specific population
(3) Validating biomarker specificity in real world settings using conventional eating patterns where a whole diet is consumed rather than focusing on single food items
(4) Evaluating the impact of food preparation/processing/formulation and cooking method on the behavior of biomarkers of specific foods
(5) Developing a urine sampling strategy that enables collection of samples with minimal burden on free-living participants and without adversely affecting the quality and comprehensiveness of biomarker measurement

The MAIN (Metabolomics at Aberystwyth, Imperial and Newcastle) Study was designed to address these challenges by investigating biomarkers of food intake under conditions in which study participants consumed well-characterized foods within conventional diets with respect to meal design, cooking and eating patterns and collected urine samples at home without changing their usual behavior. This provided an opportunity to expand biomarker coverage to include a more comprehensive range of foods and beverages that are highly consumed in the UK and considered important for the UK government healthy eating policy (25). Our primary aim was to develop protocols which could be applied in large-scale epidemiological studies, clinical trials and in public health surveys. We focused on approaches that would be acceptable to the public, easy to follow and to adhere to, and which would be of modest cost. In this paper, we report the detailed design and protocol of the MAIN Study conducted at Newcastle University (MAIN Study Newcastle) as well as baseline characteristics of participants recruited to the study. Elsewhere we have reported the validation of the urine sampling methodology for free-living study participants showing it was non-intrusive, imposed low participant burden, and delivered samples with high quality metabolome content assessed using metabolite fingerprinting (26, 27). Here, we include a summary of novel biomarkers discovered using samples from the MAIN Study with the aim of extending the BFI coverage to a wider range of commonly consumed foods.

## Materials and Methods

### Ethics Approval and Consent to Participate

The studies involving human participants were approved by East Midlands—Nottingham 1 National Research Ethics Committee (14/EM/0040) following Proportionate Review. Caldicott approval for storage of data and data protection was granted by Newcastle-upon-Tyne Hospitals NHS Foundation Trust [6896(3109)]. The trial was adopted into the UK Clinical Research Network (CRN) Portfolio (16037) and was registered with International Standard Randomized Controlled Trial Number (ISRCTN), 88921234.

A study information sheet was given to all potential participants in advance of their first visit to the research unit. The participants provided written informed consent to participate in each study, taken by an appropriately trained researcher. All procedures performed in studies involving human participants were in accordance with the ethical standards of the institutional and/or national research committee and with the 1964 Helsinki declaration and its later amendments or comparable ethical standards.

### Participant Recruitment

Participants were recruited using the inclusion criteria in Supplementary Table 1 through poster and leaflet campaigns around Newcastle University campus and in local public buildings e.g., libraries. In addition, invitation letters were sent to potential participants who had registered their interest with Newcastle University in being contacted about upcoming nutrition-related studies. An advertisement for the study was also placed in a local newspaper. Some participants were recruited by word of mouth.

Based on data from our earlier studies (28), we aimed for a sample size of 15 participants for Study 1 (which incorporated experimental period 1 menu plans) and 30 participants for Study 2 (which incorporated experimental period 2 menu plans)—this allowed for a 20% drop out. To minimize the risk that current disease or medications used for their management altered metabolism and, therefore, compromised the normal behavior of food-related biomarkers detected in urine, we implemented an *a priori* exclusion list (Supplementary Table 1). For similar reasons, we excluded potential participants who reported that they had had a cholecystectomy or who undertook a high level of exercise such as a professional athlete or body builder. Vegetarian recruits had to be willing to eat meat and fish during the studies.

### Intervention Design and Randomisation

The MAIN Study Newcastle was built around six daily menu plans, delivered in two separate, 3-day experimental periods. Menu plans 1–3 constituted experimental period 1; menu plans 4–6 constituted experimental period 2. Menu plans were designed to emulate real world conditions and to reflect the whole diet by including many commonly-eaten foods in the context of a typical UK diet. All foods and drinks were provided to free-living individuals who prepared and consumed the meals in their own homes/places of work and were responsible for collecting urine samples while carrying out their normal daily activities.

Experimental period 1 menus were based largely on foods for which a significant amount of metabolite-based biomarker research has been published. The menus permitted the provision of 4–5 target foods each day, providing opportunity for biomarker validation as described in Lloyd et al. (27). On the first experimental day, participants consumed test foods for which there were some previously published urinary metabolite biomarkers while, on the two subsequent experimental days, they consumed foods for which putative biomarkers have been proposed but for which further validation and/or discovery is necessary. This first experimental period allowed the refinement of a spot urine sampling protocol for use in real world settings. Participants collected a range of post-prandial spot urines, which were compared for chemical richness and evaluated for the presence of known and putative dietary biomarkers. The overarching aims of this part of the study were to: (i) identify the urine collection times which provided the most data-rich spot urine for accurate capture of biomarker behavior relating to recent dietary intake and (ii) develop acceptable, non-onerous and easy-to-implement urine sampling protocols for the future identification and validation of novel biomarkers of foods included within the menus [see Lloyd et al. (27) and Wilson et al. (29) for further detail].

**Figure 1:**
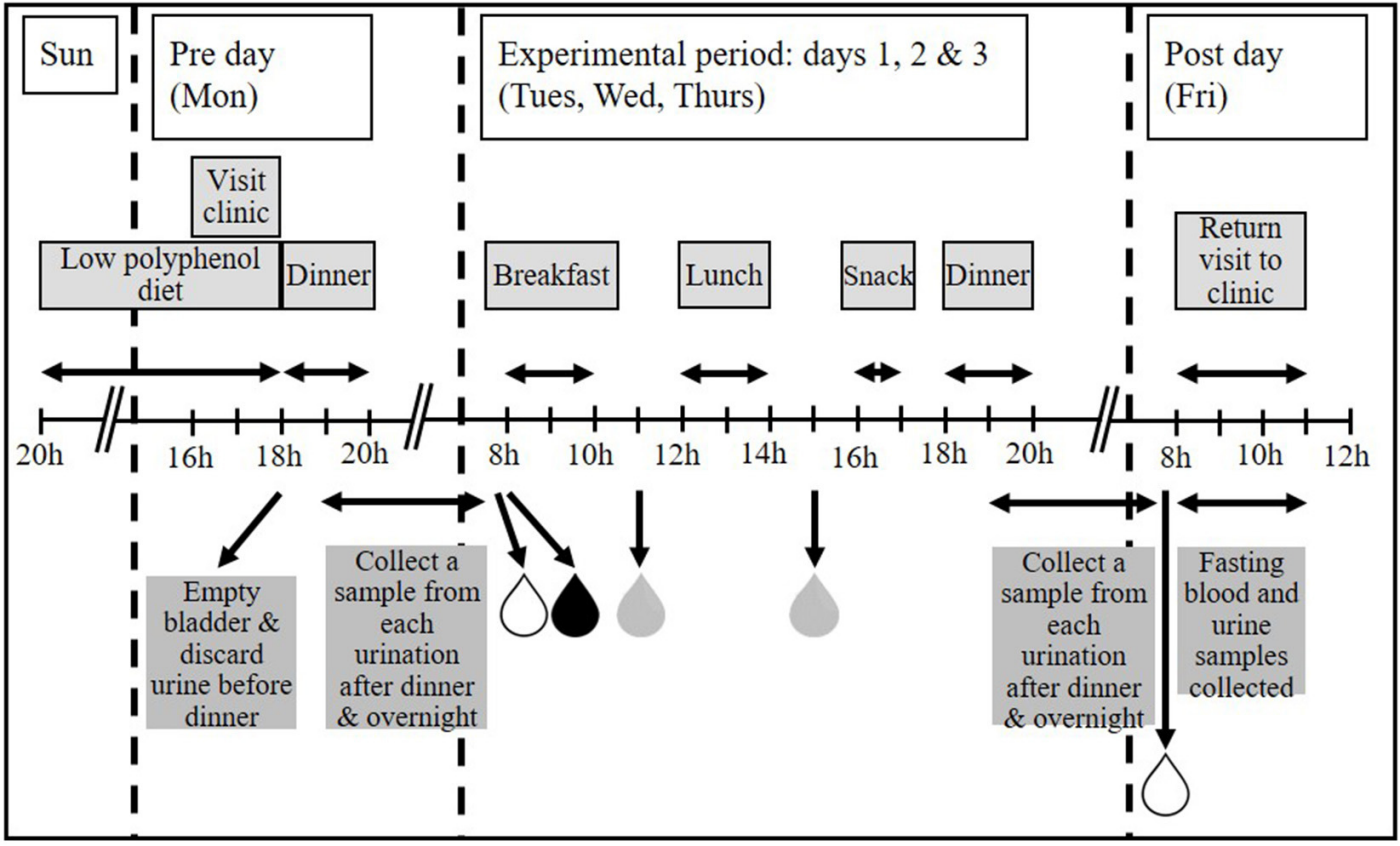
The MAIN Study timeline. Participants were asked to follow a low polyphenol diet prior to starting the experimental period. Where applicable, dinner was provided on the “Pre” day (Monday). All foods and drinks for 3 consecutive experimental days (Tuesday, Wednesday, and Thursday) were provided. Participants collected first morning void (FMV), fasting urines and spot urines post-breakfast and post-lunch each day during the experimental period, and a FMV and fasting urine on the “Post” day. Any urination between finishing dinner and providing the FMV the following day was also collected. A fasting blood sample was taken on the “Post” day only. Figure adapted from Lloyd et al. (27).

To assess whether use of a standardized evening meal [as employed in our earlier studies (30)] was advantageous for “normalizing” urines prior to biomarker validation and discovery, participants followed these menus in the same order on 2 separate weeks. Participants were randomized to either a standardized evening meal (chicken casserole ready meal and chocolate éclair) or a low polyphenol evening meal of their choice, to be eaten immediately before the experimental period. Participants were provided with a guide to high polyphenol foods/drinks that they should not eat/avoid and a list of low polyphenol foods/drinks which they should select from during the “Pre” day (see Figure 1). A cross-over design was employed so that all participants underwent both dietary interventions i.e., standardized evening meal and own choice low polyphenol evening meal.

Experimental period 2 menu plans were designed to identify novel biomarkers of food intake and to investigate whether the putative urinary biomarkers for specific foods were influenced by food preparation, processing, quality, formulation, complexity, and cooking method. Menus were created to deliver foods for which there were few, uncertain or no published biomarkers. In addition, we expanded the range of food formulations investigated in experimental period 1. For example, red and white grapes were included in the following forms: whole grapes, raisins (dried), wine (fermented), grape juice (pasteurized/heat-treated/concentrated), sparkling grape juice (carbonated drink), and a fruit smoothie (complex beverage). Whole grains were delivered across both experimental periods in the form of rye bread (with and without a sourdough starter, toasted and untoasted), wholemeal bread (toasted and untoasted), wholegrain breakfast cereal (Weetabix®), porridge oats (boiled/microwaved with milk) and wholemeal pasta (extruded). Participants consumed a range of commonly eaten foods in three different daily menu plans, which were presented in a different order in each of three experimental weeks. This design facilitated the search for novel biomarkers of specific foods, but also provided the potential to characterize the kinetics of biomarker appearance and decay and to investigate the longevity of biomarker signals in urine.

Each daily menu plan was designed to emulate conventional UK eating patterns with a breakfast, lunch, afternoon snack and dinner. All foods and drinks for the whole intervention were provided to participants in appropriate portion sizes and with cooking instructions, where necessary. Participants were encouraged to consume these items to the exclusion of any other foods or beverages, but had the freedom to eat or not the meals and to interpret the cooking instructions as they wished. During each experimental week, the allocated menu plans were followed from Tuesday to Thursday, as shown in Figure 1. Experimental period 1 menu plans were eaten in the same order on 2 consecutive weeks (Study 1); experimental period 2 menu plans were eaten in pre-determined orders over a 3-week period (Study 2). In each experimental week, the “Pre” day was the day before starting the experimental period and was always a Monday and included a pre-determined evening meal (Dinner). The “Post” day was the day following completion of the experimental period when the last biological samples were collected and was always a Friday. Participants visited the Clinical Aging Research Unit, Newcastle University on these 2 days only. This design minimized the number of trips the participants had to make to the University and also enabled participants to take a break from the studies at weekends, so reducing the burden of compliance.

During the “Pre” day, participants were asked to restrict, as far as possible, their polyphenol intake. In practical terms, this meant reducing their intake of brightly colored fruits and vegetables, chocolate, tea, herbal teas and coffee. Participants were asked to abstain from alcohol and stop taking any dietary supplements for the duration of the studies. In Study 1, dinner on the “Pre” day was either a standardized evening meal or a low polyphenol meal of the participant's choice whilst in Study 2 it consisted of one of three meals designed to capture biomarkers of relatively unhealthy food choices, particularly poorer-quality meats and breaded and battered foods.

Menu plans were developed using Public Health England policy advice from The Eatwell Plate which has been revised as The Eatwell Guide (25) and information on the eating habits of the British population which were collected and collated in the National Diet and Nutrition Survey (NDNS), years 1–3 (31). The process of menu plan design has been described in detail in a recent publication (27). Briefly, this involved analysis of each food grouping described in the Eatwell Plate (e.g., fruit & vegetables) and investigating the disaggregated food groups that contribute to that food grouping in the NDNS (e.g., fresh & canned fruit; fruit juice; dried fruit etc.). We then identified the most commonly eaten foods within each disaggregated food group and incorporated as many as possible of the most commonly consumed foods into the menu plans using the most commonly reported method of preparation (e.g., raw/boiled/with or without skin). To assess biomarker robustness, multiple forms of similar foods using different processing methods and formulations were delivered as discrete meal components and incorporated within complex meals, and using different cooking methods. Foods and portion sizes were compatible with normal eating behavior and were provided according to conventional UK daily meal patterns using commercially available foods. We used average (medium) portions of each food as determined by the Food Standards Agency “Food Portion Sizes” Guide (32). Exact amounts of each food provided has been described elsewhere (26, 27). An example daily menu plan from experimental period 2 is shown in Supplementary Table 2.

To identify the easiest to collect and most informative spot urine sample, participants were asked to collect several spot urine samples each day. These included their first morning void (FMV) before breakfast; a fasting sample, defined as a sample collected after the FMV but before breakfast, following an overnight fast of at least 12 h; a sample collected any time between finishing breakfast and eating lunch and a sample collected any time between finishing lunch and eating their afternoon snack. Each urination between dinner and the FMV the following day was also collected. To reduce variation in timing of urine sample collection, participants were encouraged to consume meals within a 2 h time slot (breakfast, lunch and dinner) or 30 min time slot (afternoon snack) as illustrated in Figure 1. However, the participants were free to ignore this advice and to provide a urine sample at the time most convenient for them.

Participants were encouraged to keep hydrated and to drink water *ad libitum*. To help maintain good hydration, participants were given eight 500 ml bottles of water per experimental week, one to be drunk with/after each dinner, one to be drunk during each experimental day and one to be drunk on the morning of the “Post” day, prior to venipuncture.

Participants in Study 1 were randomized (random.org) at enrolment to one of two dinner options on the “Pre” day. In Study 2, participants were randomized to one of 12 3 × 3 Latin squares (3 daily menu plans × 3 experimental weeks) at enrolment. The order in which these participants consumed the three less healthy dinners on the “Pre” day in each of the three experimental weeks was randomized independently.

### Compliance

Dietary compliance was assessed by participant self-report. They were asked to record how much of each food/drink item they ate. If some food was uneaten, the participant recorded the amount eaten as 75, 50, 25% or 0, as appropriate. Dietary substitutions, intrusions and protocol deviations were also recorded. Participants recorded if they substituted any foods or drinks of their own choosing for those provided by the research team, ate or drank any additional items or prepared any of the meals differently from that instructed.

A 1-day food diary was used to record all foods and drinks consumed from 8pm on the evening before and throughout the “Pre” day. This was used to check participant compliance with the low polyphenol diet prior to commencement of the experimental period each week.

To monitor compliance with both the urine collection protocol and suggested meal times, participants completed a Urine sample collection record and a Meal time record—the latter asked specifically at what time they finished eating each meal. This acted as a check on the accuracy of the FMV and fasting urine samples collected. In addition, this information allowed calculation of the time interval between meal consumption and subsequent urine collections.

### Study Measures

Information on participant socio-demographics (age, sex, smoking status, and alcohol consumption), medical history, current medications, and use of dietary supplements was collected at enrolment.

In Study 1, weight, height and waist circumference were measured on “Pre” and “Post” days of each experimental week. In Study 2, height and waist circumference were recorded in week 1 only, whilst weight was recorded weekly, pre- and post- the experimental period. All anthropometric measurements were made at the research unit by the same researcher.

Body weight was measured to the nearest 0.1 kg using a Tanita body composition analyzer (TBF-300 MA; Tanita Corporation, Tokyo, Japan); height was measured to the nearest 0.1 cm using a Leicester portable height measure (Chasmors Ltd, London, UK). Waist circumference (at the point equidistant between the costal margin and the iliac crest) was measured to the nearest 0.1 cm using a non-stretch tape measure over bare skin, whenever possible. Waist circumference measurements were taken in duplicate, or repeated until two measurements agreed within 1 cm. Participants were asked to wear lightweight clothing and removed their shoes for all measurements.

Habitual dietary intake was assessed at the beginning of each study using a locally adapted version of the validated food frequency questionnaire (FFQ) used in the European Prospective Investigation into Cancer and Nutrition (EPIC) (33).

To monitor their physical activity (PA), each week participants were asked to complete the International Physical Activity Questionnaire (IPAQ) short form (34).

### Blood and Urine Sample Collection

Participants collected urine samples at suggested times (Figure 1) in a calibrated plastic jug and recorded the date, time and total volume of collection. A 20 ml aliquot from each urination was retained and the rest discarded. If not at home, participants kept urine samples in a cool bag containing a frozen cool block, otherwise they stored them in a refrigerator before returning them to the research team at the end of each experimental week. It has been reported that no major changes in urinary metabolite fingerprints occur when samples are stored in tubes held at +4°C for up to 72 h (35). We have shown recently that the metabolome of spot urine samples collected, stored and transported as described in this manuscript is stable with negligible microbial growth at 4°C and, specifically, that inclusion of preservatives has no impact on data quality (36). For long-term storage, urine samples were divided into 10 × 1 ml aliquots [2 ml screw-cap microtubes (Starstedt, Germany)] which were free from plasticisers (in house tests; data not shown)] and 1 × 5–10 ml aliquot (25 ml Universal tubes; Starstedt) and stored at −80°C.

Whole blood, serum and plasma were archived at −80°C for future lipidomic analysis and associated metabolite fingerprinting. Venipuncture was performed on the “Post” day of each week after a minimum 12 h overnight fast. After filling, blood tubes were inverted several times, kept flat on ice and processed within 2 h. Before processing, the height of the blood in each tube was recorded to assess filling. Plasma was separated from anti-coagulated blood collected in 1 × EDTA and 2 × lithium/heparin Vacutainer® blood collection tubes (Becton Dickinson, Oxford, UK) by centrifugation at 3,100 × g for 5 min at 4°C in a Jouan CR3i centrifuge (Saint-Herblain, France). Plasma was stored at −80°C in 1 ml aliquots in plasticiser-free microtubes as described above. Serum was separated from coagulated blood collected in 2 × gel Vacutainer® tubes at least 30 min after collection, aliquoted and stored as for plasma. One tube of unprocessed whole blood, collected in an EDTA-coated tube, was also stored at −80°C.

A summary of the time points at which key data, measurements and biological samples were collected is given in Table 2 (Study 2) and Supplementary Table 3 (Study 1).

**Table 2:** Summary of data and biological samples collected during Study 2.

**Data collected/measures**	**Study time point**									
		**Experimental week 1**			**Experimental week 2**			**Experimental week 3**		
	**Screening**	**Pre day visit**	**Between visits**	**Post day visit**	**Pre day visit**	**Between visits**	**Post day visit**	**Pre day visit**	**Between visits**	**Post day visit**
Demographics (age, sex) & self-reported anthropometrics	X									
Eligibility criteria (medical history, medications, supplements, diet & lifestyle)	X									
Written consent		X								
Randomisation		X								
One day food diary		X			X			X		
Height & waist circumference		X								
Weight		X		X	X		X	X		X
Food frequency questionnaire		X								
IPAQ Physical activity questionnaire		X			X			X		
Dietary compliance record			X			X			X	
Meal time record			X			X			X	
Urine samples			X	X		X	X		X	X
Urine sample collection record			X	X		X	X		X	X
Blood sample (plasma, serum & whole blood)				X			X			X

*IPAQ, International Physical Activity Questionnaire*.

### Metabolomic Analysis

The metabolomics methods implemented for biomarker discovery have been published elsewhere (26, 27). All urine samples were normalized by refractive index prior to analysis to ensure all MS measurements were made within a similar dynamic range. Essentially, urine samples were analysed by non-targeted metabolite fingerprinting using high resolution (HR) flow infusion electrospray (FIE) ionization mass spectrometry (MS), acquired on an Exactive Orbitrap (ThermoFinnigan, San Jose, CA) mass spectrometer coupled to an Accela (ThermoFinnigan) ultra-performance liquid chromatography system. Supervised Random Forest (RF) classification was implemented to investigate the presence of distinctive urine composition changes following consumption of specific meals. A combination of accuracy, margins of classification and area under the ROC (Receiver Operator Characteristic) curve (AUC) were used to evaluate the performance of classification models (37). To reveal potential explanatory signals responsible for discriminating between baseline and post-prandial urine samples, a combination of RF, AUC and Student's *t*-test was employed for feature selection (37).

The methodology used for biomarker identification has been described in detail elsewhere (26, 27). For metabolite signal annotation, accurate *m/z* values were extracted for high-ranked explanatory signals and queried using MZedDB, an interactive accurate mass annotation tool (38). Ultra High Performance Liquid Chromatography-High Resolution MS (UHPLC-HRMS) and Tandem mass spectrometry (MS^n^) allowed further structural identification of putative biomarkers as previously described (39). Refractive index adjusted urine samples were diluted with 100% MeOH (1:1, v:v) and centrifuged at 1,400 × *g* for 5 mins at 4°C. Samples were analysed on an Orbitrap Fusion Tribrid mass spectrometer (Thermo Scientific, Waltham, MA) coupled to a Dionex Ultimate 3000 Ultra High Performance Liquid Chromatography (UHPLC) system (Thermo Scientific). Chromatographic separation was performed on a reverse phase (RP) Hypersil Gold 1.9 μm, 2.1 × 150 mm column (Thermo Scientific) using 0.1% formic acid in H_2_O (mobile phase A) and 0.1% formic acid in MeOH (mobile phase B) at a flow rate of 0.6 ml/min and column oven temperature at 60°C. Each sample (5 μl) was analysed by following a gradient after 0.5 min isocratic A to 40 % B in 3.5 min and subsequently to 100% in 5 min. The column was washed with 100% B for 2.5 min and re-equilibrated for 2.5 min. Data were acquired in two runs using respective positive and negative ESI mode. Each experiment consisted of a full scan [110–1,100 *m/z* at 120,000 resolution and MS^2^ scans (*ddMS2 OT HCD* event, stepped Higher-energy collision energies of 45, 60, 75%) and 15,000 Orbitrap mass resolution] within a 1 s cycle time using selected targeted mass properties for either positive or negative ionization mode between 1 and 12 min runtime. The maximum injection time was 22 ms and the Automatic Gain Control (AGC) target of 1 × 10^4^ was set to be exceeded if there is parallelisable time. The spray voltage was 3.5 kV for positive and 2.5 kV for negative ionization modes. The temperatures of the ion transfer capillary and vaporiser were, respectively, 342° and 258°C with sheath and auxiliary gas set at 45 and 13 arbitrary units, respectively. The data were acquired using Thermo Scientific Xcalibur version 4.2.28.14.

Metabolites were annotated to Metabolomics Standards Initiative (MSI) level 1 (40) by matching masses, MS^n^ and retention times with authentic standards or with the respective aglycone (if the biotransformation product was unavailable). MSI level 2 structural assignment was achieved by putatively matching signal behavior with that of authentic standards reported in the literature (based upon physicochemical properties, retention times and spectral similarity) or fragmentation pattern alignment with data in public/commercial spectral libraries [Lipid Maps, HMDB, Metlin, and Massbank (41–44)]. MSI level 3 structural identification indicated a putatively characterized compound class.

## Results

### Recruitment

**Figure 2:**
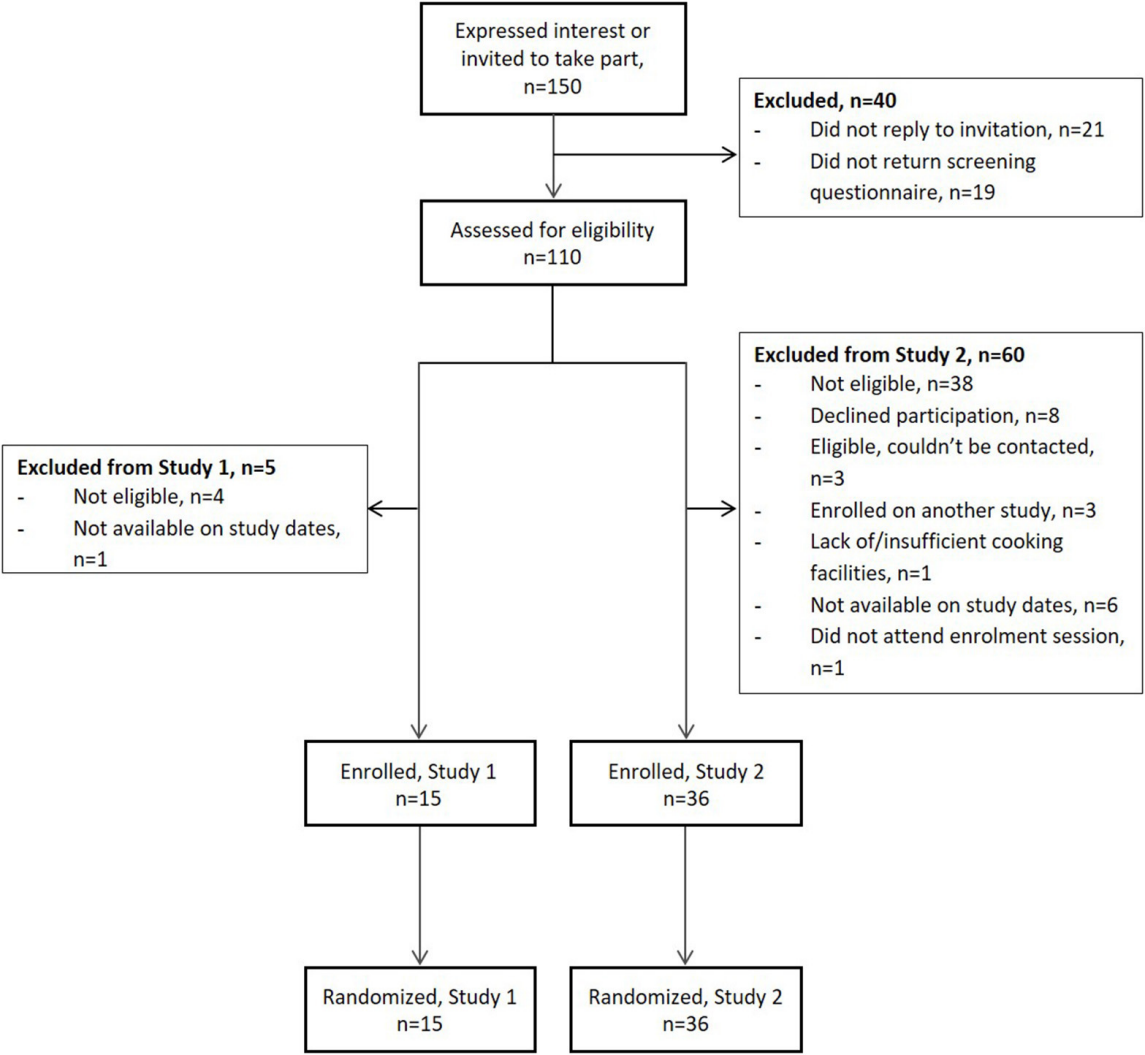
Flow of participants during recruitment to the MAIN Study. Study 1 preceded Study 2; six participants took part in both studies.

As shown in Figure 2, 150 people expressed an interest, or were invited to participate, in the studies over a 10 month period: 40 people for entry into Study 1 and 116 people for entry into Study 2 (six people took part in both studies). Personal details, self-reported anthropometrics, medical history and lifestyle information (smoking, alcohol consumption, dietary supplementation) were provided by 70% people (*n* = 105). More than 70% of those invited to participate in Study 1 had taken part in earlier nutrition research studies at Newcastle University and six were subsequently recruited. A further nine participants learned about the study through word of mouth. The majority (85%) of those who expressed an interest in Study 2 and were screened for eligibility (69%) had learned about it through local community and internal university advertising and contributed 66% of enrolled participants. The remaining participants had either taken part in Study 1 (17%) or heard about the study through word of mouth (17%). The desired sample size (*n* = 15) was met in Study 1 and the target recruitment (*n* = 30) was exceeded by 20% in Study 2.

### Baseline Characteristics of Participants

Participants aged 19–77 years (mean 46 years), of whom 57% were female, took part in the studies (Table 3). The participants were generally healthy: arthritis, previous cancer and hypertension were the most commonly reported medical history. Smokers were excluded from the study and while 78% participants were alcohol consumers, weekly consumption (median 4.5 units) was well below the recommended maximum levels. Mean BMI (24.1) was within the normal range but almost 20% of participants had central obesity. Twenty-five percent of participants reported taking dietary supplements with fish oils/cod liver oil being the most commonly consumed supplement. Self-reported physical activity levels were relatively high−59% of participants were categorized as highly physically active according to the IPAQ guidelines (45). Self-reported sitting time, measured by the IPAQ, was also high with participants reporting, on average, 6 h sitting time/day. This exceeds the 4 h sitting time/day cut-off point considered a proxy for sedentary behavior detrimental to health (46).

**Table 3:** Characteristics of the MAIN Study participants at baseline.

	**Study 1**	**Study 2**	**Total**
**Variable**	**Mean (SD)**	**Mean (SD)**	**Mean (SD)**
***Demographics***			
Total [n]	15	36	51
Sex			
Female [%]	53	58	57
Age [years]	45.3 (14.8)	46.7 (18.7)	46.3 (17.5)
Age range (min-max) [years]	22–63	19–77	19–77
***Health Conditions [n]***			
Arthritis	0	4	4
History of cancer	1	3	4
Hypercholesterolemia	0	1	1
Hypertension	0	4	4
Irritable bowel syndrome	1	0	1
Osteoporosis	0	1	1
Stomach/bowel problems	0	1	1
***Diet [n]***			
Vegetarian	0	1	1
Pescatarian	1	1	2
Food Allergies^#^	0	2	2
Did not eat pork for religious reasons	0	1	1
Supplement use	4	9	13
Sport supplement^¥^	1	0	1
Vitabiotics Osteocare^≠^	1	0	1
Guarana	1	0	1
Ginkgo Biloba	1	0	1
Aloe Vera	0	1	1
Garlic	1	1	2
Evening Primrose oil	1	1	2
Fish oils/cod liver oil	1	6	7
Glucosamine	0	2	2
Chondroitin	0	1	1
Zinc	0	1	1
Cranberry extract	0	1	1
Vitamin D	0	1	1
Vitamin C	0	1	1
Multivitamins	0	1	1
***Lifestyle^a^***			
Alcohol			
Consumers [%]	87	75	78
Consumption [units/wk]*^b^*	4 (2.5–9)	6.5 (2–13.5)	4.5 (2–11)
***Anthropometrics***			
Weight [kg]	71.9 (13.5)	66.8 (10.4)	68.3 (11.5)
Height [cm]	169.5 (9.0)	167.6 (7.7)	168.2 (8.0)
Waist circumference [cm]	84.5 (11.3)	82.5 (9.1)	83.1 (9.7)
BMI [kg m^−^^2^]*^c^*	24.9 (3.7)	23.7 (3.1)	24.1 (3.3)
Weight Status [%]			
Normal	60.0	66.7	64.7
Overweight	33.3	27.8	29.4
Obese	6.7	5.6	5.9
Central Obesity*^d^*	26.7	16.7	19.6
***Physical Activity^e^***			
Total PA [MET-mins/week]	2,747 (1,969–5,058)	2,994 (1,866–4,878)	2,937 (1,894–4,878)
Activity Level [%]			
High	69.2	54.8	59.1
Moderate	30.8	29.0	29.5
Low	0	16.1	11.4
Sitting time [mins/day]*^f^*	390 (285–480)	360 (240–435)	360 (255–480)

*In this table data are presented as mean (standard deviation, SD) for continuous variables and as number of participants or percentage (%) for categorical variables, except for alcohol consumption, total physical activity (PA), and sitting time, which are given as median (interquartile range). MET, metabolic equivalent of task*.

a *smokers were excluded from the study*.

b *participants who reported no alcohol consumption were excluded from this analysis*.

c *body Mass Index (BMI) was calculated as [weight(kg)/height(m)2]. BMI cut-off points for determination of weight status were: normal weight 18.5–24.9 kgm−2, overweight 25.0–29.9 kgm−2, obese 29.9–39.9 kgm−2*.

d *central obesity was determined using waist circumference as a proxy, with sex-specific cut-off points (females ≥ 88 cm, males ≥ 102 cm)*.

e *to classify individuals according to their self-reported PA, MET-minutes per week were calculated and participants were grouped into three activity levels (high, moderate, low) according to the cut-points defined in the International Physical Activity Questionnaire (IPAQ) guidelines (45). Outliers were excluded along with datasets of individuals containing missing values (n = 2). Reported duration of activity was truncated to 180 min, where necessary, according to the IPAQ data processing rules*.

f *sitting time is defined as a sedentary-related behavior (47) and spending 4 h or more (≥240 min) a day sitting is a proxy measure of sedentary behavior detrimental to health (46)*.

# *food allergies were to shellfish (1 person) and whole egg/milk (1 person)*.

¥ *each serving contains 150 mg of caffeine and 1.7 g creatine monohydrate, plus specific amino acids, vitamins, fruit extracts, and black pepper extracts*.

≠ *contains calcium, vitamin D, zinc, and magnesium*.

Participant characteristics were similar in both studies but those recruited to Study 2 were slightly older (mean 1.4 years) and had a lower BMI. They were less likely to report consuming alcohol, although weekly consumption amongst those who did was higher (based on self-reported weekly alcohol intake). Ten percent more participants were categorized as having central obesity in Study 1 than in Study 2.

### Validation of Study Design for Discovery of Novel Biomarkers of Dietary Exposure

In recent publications, we have reported that dietary exposure biomarker discovery was possible within the context of the present comprehensive food intervention mimicking a typical UK diet in free-living individuals with minimal intrusion on normal daily activities (26, 27). Validation of the overall MAIN Study Newcastle design for efficient biomarker discovery was assessed initially by confirming the presence of expected BFIs in urine samples collected during and following experimental day 1 in Study 1 (26). Biomarker performance was further tested using different food formulations and processing methods with several types of meat, wholegrains, fruit and vegetables (26). Additionally it was shown that the urine sampling methodology for free-living study participants was non-intrusive and delivered samples with high quality metabolome content using metabolite fingerprinting (27).

**Figure 3:**
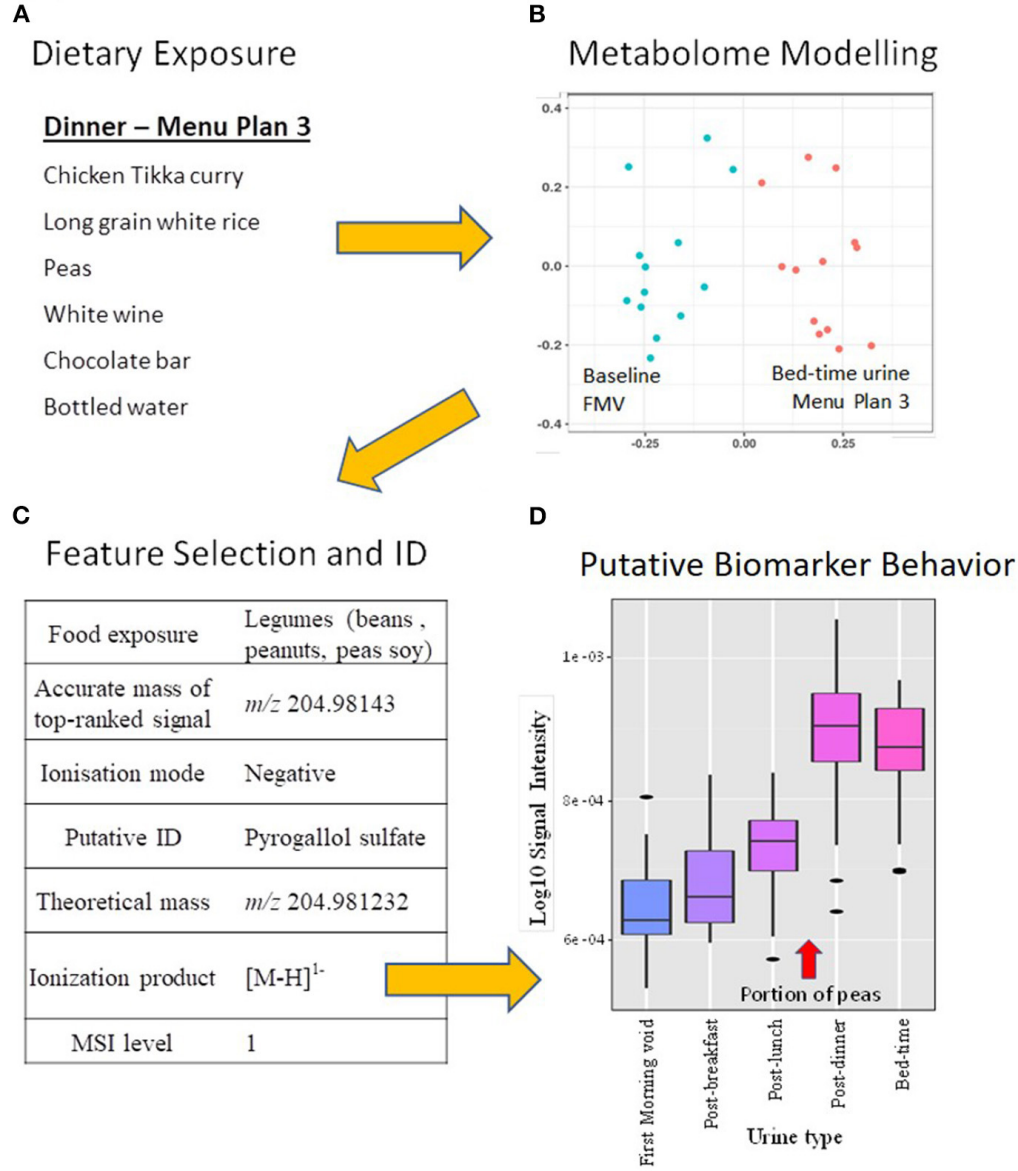
Schematic of the dietary exposure biomarker discovery strategy within the context of a comprehensive food intervention mimicking a typical UK diet in free-living individuals. **(A)** Meal items consumed at Dinner time on Menu plan 3 [details in Lloyd et al. (27)]; **(B)** Multi-dimensional scaling (MDS) of Random Forest (RF) proximity values of the FIE-HRMS urinary fingerprint data of first morning void and bed-time urines from the same day that Menu plan 3 was consumed; **(C)** Annotation of a metabolite signal highly explanatory of legume exposure on several experimental days when legumes were included on the menu; **(D)** Box-plots showing the association between pea consumption and the relative intensity of the pyrogallol sulfate signal [M-H]^1−^ in urine samples taken throughout the day that Menu plan 3 was eaten.

Against this background, we now demonstrate that the MAIN Study Newcastle design, coupled with metabolomic techniques, made possible the discovery of new BFIs for use in monitoring dietary intake in free-living individuals eating conventional diets. As an example, the schematic of the dietary exposure biomarker discovery strategy using BFIs of legumes is shown in Figure 3. Participants were exposed to an evening meal (Figure 3A) containing a typically consumed legume (garden peas). Random Forest modeling of metabolite fingerprints representing urines collected at bed-time following this meal and FMV urine samples acquired earlier in the day showed that the urine samples had very distinctive compositions (Figure 3B). Feature selection identified several metabolite signals that were strongly explanatory of compositional differences between these two urine classes. Further analysis of urine composition after consumption of a range of legumes (beans, soy and peanuts) in other MAIN Study Newcastle menu plans revealed several explanatory metabolites in common, including *m/z* 204.98143. Detailed structural analysis indicated that this particular compound is pyrogallol sulpate (Figure 3C). The large relative increase in the pyrogallol sulpate signal after consumption of peas in shown in Figure 3D.

Following an identical rationale, a selection of potential biomarkers of an extended range of foods is summarized in Table 4. In addition to pyrogallol sulfate, pyrogallol glucuronide and trigonelline emerged as generic biomarkers of legume consumption (beans, peanuts, peas and soy). Eugenol glucuronide and eugenol sulfate were elevated in urine after consumption of curry and are potential biomarkers of this food group. The metabolite, 2-furoylglycine, appeared discriminatory for high temperature baked foods (e.g., pies) and toasted grain products (e.g., toasted bread). We identified furaneol (sulfate and glucuronide) and mesifurane after the consumption of strawberries and tomato products. Higher concentrations of Maillard reaction intermediates 2,4-dihydroxy-2,5-dimethyl-3(2H)-furanone (acetylformoine) in both its sulfate and glucuronide forms and norfuraneol sulfate (4-hydroxy-5-methyl-3(2H)-furanone) were observed in urine following consumption of both high temperature-baked and toasted grain products as well as strawberries, berries and tomato. After the consumption of a low-calorie beverage, urinary concentration of the sweetener acesulfame potassium was elevated for up to 12 h (Table 4).

**Table 4:** Discovery of novel biomarkers for foods where biomarkers have yet to be discovered in relation to UK Public health policies.

**Food exposure source**	**Biomarker**	**Ionization products**	**MSI level**
Legumes	Pyrogallol (1,2,3-Trihydroxybenzene) glucuronide	[M-H]^1−^	1
	Pyrogallol (1,2,3-Trihydroxybenzene) sulfate	[M-H]^1−^, [M-H]^1−^^13^C, [M-H]^1−^^34^S	1
	Trigonelline	[M+H]^1+13^C, [M+Na]^1+^, [M+Na]^1+^ ^13^C, [M+K]^1+^, [M+K]^1+13^C, [M+K]^1+41^K	1
Curry (clove)	Eugenol glucuronide	[M-H]^1−^, [M-H-gluc]^1−^, [M-H-gluc]^1−13^C	1
	Eugenol sulfate	[M-H]^1−^, [M-H]^1−34^S	1
High temperature baked and toasted grain products	2-Furoylglycine	[M+Na]^1+^, [M+K]^1+^, [M+2Na-H]^1+^, [M+KNa-H]^1+^, [M-H]^1−^	1
Strawberry, berries, and tomato	Furaneol sulfate	[M-H]^1−^, [M-H]^1−13^C, [M-H]^1−34^S	1
	Furaneol glucuronide	[M-H]^1−^	1
	Mesifurane (2,5-Dimethyl-4-methoxy-3(2H)-furanone) sulfate	[M-H]^1−^	2 (48, 49)
High temperature baked and toasted grain products and strawberry, berries, and tomato	Norfuraneol sulfate (4-hydroxy-5-methyl-3(2H)-furanone)	[M-H]^1−^, [M-H]^1−34^S	1
	2,4-Dihydroxy-2,5-dimethyl-3(2H)-furanone sulfate	[M-H]^1−^	3
	2,4-Dihydroxy-2,5-dimethyl-3(2H)-furanone glucuronide	[M-H]^1−^, [M-H]^1−13^C	3
Low calorie drinks	Acesulfame potassium	[M-K]^1−^, [M-K]^1−13^C, [M-K]^1−34^S	1

*MSI level 1, matching masses, MS^n^ and retention times with authentic standards or with the respective aglycone; MSI level 2, structural assignment achieved by putatively matching signal behavior with that of authentic standards reported in the literature or fragmentation pattern alignment with data in public/commercial spectral libraries; MSI level 3, structural identification indicated a putatively characterized compound class*.

## Discussion

### Success of Free-Living Study Design

Our aim was to design and implement a prolonged dietary intervention study which would allow the collection of urine samples for biomarker studies in a home or work environment. Study recruitment achieved (Study 1) or surpassed (Study 2) original expectations with 51 healthy adults consuming specific foods and beverages, provided *in toto* by the research team. Apart from a visit to the research unit to pick up food at the beginning, and to deliver urine samples at the end, of each experimental week, participants were free-living and had the freedom to eat or not the meals and to interpret the cooking instructions as they wished. A combination of no drop outs and high compliance rates with regard to meal consumption (> 80%), suggests that this food intervention strategy was highly acceptable to participants [see Lloyd et al. (27) for details on compliance].

Participants collected urine samples within pre-specified time frames each day and fasting blood samples were collected in the morning of the final (“Post”) day of each experimental week. This aspect of the study was designed to be as minimally intrusive as possible to investigate the potential for such urine collections to be incorporated into future larger-scale epidemiological studies and surveys. Data associated with urine collection has been described in a recent publication (27) and indicated that the most successful urine type collected within this cohort was the FMV and the post-dinner spot sample (both at 99% compliance). The fasting sample was the least successfully collected sample. Evidence that the urine sampling methodology imposed low burden on participants and delivered samples with high quality metabolome content has been published (27). From a metabolomics perspective, the overall experimental design was validated by confirming the presence of known BFIs in urine samples after exposure to menu plan 1 (26).

### Discovery of Novel Exposure Biomarkers

For BFIs to have utility in assessing dietary intake as a whole, it is essential that the dietary exposure biomarker panel is as comprehensive as possible. To date, the focus on BFI discovery has centered largely on healthy foods of high public health significance (50, 51) rather than more unhealthy foods containing high levels of fat, sugar and salt (52–54). We used our intervention design in a free-living population to aid the discovery of novel biomarkers to help complete coverage of the UK Eatwell Guide (25) whilst aiming to monitor comprehensively both the whole diet and the range of cooking methods used in populations.

UK government policy recommends the consumption of more beans and pulses and less red and processed meat (25). However, there is very limited data on potential urinary biomarkers for non-meat protein-rich foods, such as beans, lentils, and other pulses (55, 56). Here we propose pyrogallol sulfate and glucuronide as potential markers of overall legume consumption (beans, peanuts, peas, soy). Additionally, trigonelline, despite being well-documented as a coffee (57) and pea consumption biomarker (58), and most recently, a novel candidate marker for soy (59), is, in fact, a general legume BFI. However, whilst potential BFIs for legumes, these markers are not exclusive to pulses. Pyrogallol is present in low quantities in beer (60), cocoa and coffee and is excreted as a sulfate after green tea and nut consumption (61, 62). These findings illustrate the need to utilize urine samples from a comprehensive food intervention to investigate specificity to individual foods or food groups. If the dietary source(s) of trigonelline needed to be identified further, then the relative contribution of coffee and soy consumption could be estimated using additional discriminatory biomarkers such as caffeine and daidzein sulfate, respectively (57, 63). These biomarkers could be added to a panel of BFIs to monitor protein-rich food intake, together with anserine and TMAO (Trimethylamine-N-oxide) to indicate poultry and fish intake and carnitine and carnosine to indicate red meat consumption (16, 64).

It still remains challenging to assess the overall “quality” of meat products that are consumed since processed meat products have very variable levels of striated muscle content (19, 65). However, it is possible that BFIs of meal components strongly associated with generally unhealthy diet patterns (66) such as a deep fried-potato (22) or mechanically recovered meat could be highly informative (19, 65). These issues will need to be addressed in the future to provide a comprehensive panel of biomarkers that can characterize and quantify eating habits *in toto*.

Concentrations of eugenol glucuronide and eugenol sulfate in urine increased after the consumption of curry possibly because clove (a common component of curry) is rich in eugenol (67). Having biomarkers that provide information about the cooking and processing methods used with foods would be an important addition to biomarkers that reflect the raw ingredients of the dish/meal because different cooking methods can change the quality and therefore healthiness of a food. Obtaining this level of information can be difficult using self-reported dietary assessment instruments, especially widely-used food frequency questionnaires. To date, a few such biomarkers of high-temperature cooked meats have been described (68). In the present study, the marker 2-furoylglycine appeared discriminatory for thermally treated foods including pies, grains and toasted wheat products (e.g., toasted bread). This marker has been described as an acute coffee consumption biomarker (69) which results from furan metabolites that arise through roasting of coffee beans via the Maillard reaction. Our metabolomics approach identified several other thermally treated/heated food Maillard products—furanones—including 2,4-dihydroxy-2,5-dimethyl-3-furanone sulfate and glucuronide and norfuraneol sulfate, which have been detected as aglycones in bread crust and popcorn, respectively (70, 71), but not reported previously in urine samples. 2,4-dihydroxy-2,5-dimethyl-3-furanone sulfate and glucuronide have been reported as markers for the intake of deep-fried potatoes, but, due to their presence in other foods, these compounds are not likely to be specific (22). Although furanones are formed by the Maillard reaction during the thermal treatment of food, they can also be biosynthesized by plants, microorganisms, and insects [as reviewed by Slaughter (72)]. Other metabolites structurally identical to Maillard products, including furaneol and its methyl-ether derivative mesifurane, are well-documented natural aroma components in fruits such as pineapple, raspberry, mango, grapefruit, tomato, and strawberry (73) and the glucuronide and sulfate of these compounds have been reported in urine after strawberry consumption (48, 74). In the current study, we confirmed this observation and suggest that furaneol glucuronide and sulfate, and mesifurane sulfate, are generic biomarkers of berry and tomato consumption (Table 4). Assessment of the relative concentrations of some of these chemicals and strawberry/berry/tomato specific compounds {i.e., pelargonidin [strawberry], hydroxyphenylvalerolactone [procyanidin-rich food (75)] and lycopene in plasma for tomato (76)}, could provide rich information on cooking methods and could, potentially, distinguish between berry fruit and tomato consumption.

Foods high in added sugar are not needed in the diet and should only be consumed in very small amounts (25). Urinary sucrose has previously been shown to be a marker of acute sugar exposure (53) and appeared discriminatory of the consumption of high sugar products, such as sweetened breakfast cereals, in the present study (26). Many food manufacturers are now substituting sugars in traditionally sugar-sweetened beverages with low-calorie sweeteners, to help combat risks associated with high sugar intake (77). The urinary concentration of the sweetener acesulfame potassium was elevated after the consumption of a low-calorie beverage and remained so for up to 12 h after consumption (Table 4). This low-calorie sweetener is a known component of the chosen beverage and, in addition to other commonly consumed sweeteners, is a potential biomarker of recent low-calorie beverage intake (78).

### Strengths and Limitations of Study Design

The MAIN Study Newcastle is one of the largest food interventions reported to date using metabolomics approaches to discover new, and to help validate existing, biomarkers of foods frequently consumed in the UK. A key objective was to design an efficient and acceptable intervention strategy that would expose study participants to foods encountered commonly in the UK diet. We established a successful food exposure strategy using information on consumption frequencies, food groupings and eating habits from the UK NDNS data (31) together with Public Health England policy advice (25) using standard portion sizes (32). We demonstrated that it is possible to design menus that mimic major features of a typical UK diet and are suitable for short-term randomized controlled dietary interventions “experimental periods” lasting only 3 days. Importantly each experimental period contained menu plans organized to emulate conventional UK eating patterns with a breakfast, lunch, afternoon snack and dinner that differed each day. This intervention strategy may be of value for the design of future studies of BFI discovery and validation in other populations globally. This design differs from many other reported biomarker discovery studies that utilized either single meals/ingredients in isolation (15, 30, 79, 80) or repeated menus (81) or depended on supplementation of the habitual diet (82, 83). Additionally, the overall menu design ensured that participants were exposed to several formulations of individual foods. In other work within the MAIN project, we have demonstrated the value of spot urines for assessing dietary intake (29). The present protocol required all study participants to store 20 ml urine samples in their home fridges for up to 5 days before transporting them to the research unit at the end of each experimental week. We have checked that urine samples collected at home and stored at 4°C are stable and not subject to microbial degradation (36). When outside the home, small cool bags were supplied for temporary urine storage. All urine spot samples were collected with success rates exceeding 90% showing that this approach to urine sampling for biomarker studies is highly acceptable. Additionally, the collection of multiple spot urines allowed us to investigate systematically the utility of home collected spot urine samples taken at various times in the diurnal feeding and fasting cycle for BFI discovery [see Lloyd et al. (27)]. Based on these observations we suggest that the protocols used in the MAIN study have overcome many of the design challenges summarized in Table 1.

From a biomarker discovery perspective, an important finding from Study 1 was that the consumption of a standardized evening meal prior to the experimental period had little impact on the ability to discover BFIs [see Lloyd et al. (26)]. This finding simplified the design of the wider food intervention in Study 2 and reduced the burden on the study participants by requiring only a limit on the consumption of polyphenol-rich foods prior to each experimental period. Furthermore, the characteristics of the MAIN study participants (Table 3) in respect of sex, age, adiposity, physical activity and general health indicate that BFIs discovered in this study are likely to be generalizable to the wider population. Importantly, in the MAIN Study Newcastle, all foods and beverages were prepared and consumed by free-living participants in their own homes rather than in a controlled clinical facility (24). This strategy ensured that metabolomic analyses were undertaken within the context of normal eating behaviors and in real world meal patterns, rather than following consumption of discrete items in isolation. In addition, this study design took into account the inherent variability associated with unsupervised individuals preparing meals and eating them in their own homes and collecting urine samples within flexible time ranges. Importantly, the comprehensiveness of this food intervention provides opportunity to examine the specificity of putative biomarkers in relation to exposure to a wide range of foods within the same biobank of samples.

Although the MAIN study had great value for the discovery of putative BFIs, some sources of variability were not considered. For example, the study employed relatively healthy individuals and we excluded those with current disease or disease treatment which might have altered the metabolism of ingested foods. As a consequence, future studies should explore the robustness of biomarkers of food intake in population groups with poorer health, particularly those who are taking prescribed medications that affect the P450 enzyme consortium or the gut microbiome. Our cohort of participants included one individual with irritable bowel syndrome (IBS)—contrary to the study inclusion criteria—but it seems unlikely that this would have had a major effect on the overall findings. Similarly, our cohort included three participants who were slightly obese (BMI 32.2–33.0 kg/m^2^) and, again, it is unlikely that this infringement of the exclusion criteria will have affected the findings. It is possible also that participant genotype will alter the pattern of metabolites produced from a given food constituent and so for this reason further work should investigate the impact of common variants in the genes encoding Phase 1 and Phase 2 enzyme systems, an example being the P450 consortium (14), on urinary BFIs. It should be noted that several of the putative BFI reported here are biotransformation products (sulfates and glucuronides) that could be identified to the level of the aglycone only (i.e., pyrogallol, eugenol, furaneol), because chemical standards for the biotransformed products are not available commercially at a reasonable cost.

### Future Work

In previous publications, we have shown that when using a combination of non-targeted metabolite profiles and targeted BFIs for assessment of dietary patterns, spot samples are suitable replacements for 24-h urine samples (29). The collection of multiple spot urine samples throughout the day using a home urine sample collection method will enable us to determine which samples (e.g., FMV or bed-time) or combinations thereof, and how many samples, are optimal for assessment of eating behavior using BFIs. It is anticipated that multiple, well-spaced spot samples collected over several weeks would be able to capture biomarker data accurately, reflecting habitual exposure to a wide range of food groups in a similar way to that achieved using multiple 24 h recall methods. The ultimate aim of our studies is to deploy a comprehensive BFI panel to aid in monitoring habitual dietary exposure in clinical trials or population surveys at a range of scales. With this aim in mind, we have developed methodology for urine collection using vacuum transfer technology which is suitable for routine use and may provide a scalable, cost-effective means to collect urine samples and to assess dietary intake in large-scale epidemiological studies and in public health surveys (36).

## Data Availability Statement

The raw data supporting the conclusions of this article will be made available by the authors, without undue reservation.

## Ethics Statement

The studies involving human participants were reviewed and a favorable ethical opinion was obtained following Proportionate Review by the East Midlands—Nottingham 1 National Research Ethics Committee (14/EM/0040). Caldicott approval for storage of data and data protection was granted by Newcastle-upon-Tyne Hospitals NHS Foundation Trust [6896(3109)]. The trial was adopted into the UK Clinical Research Network (CRN) Portfolio (16037) and was registered with International Standard Randomized Controlled Trials Number (ISRCTN), 88921234. A study information sheet was given to all potential participants in advance of their first visit to the research center. Written informed consent was obtained prior to participation from each eligible individual, for each study, by an appropriately trained researcher. All procedures performed in studies involving human participants were in accordance with the ethical standards of the institutional and/or national research committee and with the 1964 Helsinki declaration and its later amendments or comparable ethical standards. Trial registration: ISRCTN, ISRCTN88921234. Registered 3rd April 2014—retrospectively registered, http://www.isrctn.com/ISRCTN88921234. The patients/participants provided their written informed consent to participate in this study.

## Author Contributions

JD, JM, and MB conceived the study. JM, JD, NW, AL, and MB designed the study and menu plans. NW and LX undertook participant recruitment, developed participant handling protocols, ran the intervention study, supervised support staff, collected all biological samples, and refined sampling methodology. IG-P and EC coordinated project input from Imperial College London. NW and MS analysed and interpreted the data with respect to study design, participant recruitment and characteristics and participant and sample handling, and produced the tables and figures. KT and MB analysed the urine samples. AL analysed and interpreted the metabolomics data and produced the tables and figures. NW, AL, JD, and JM wrote the manuscript. JM and JD coordinated the project and supervised the research teams in Newcastle and Aberystwyth Universities, respectively. All authors read and approved the final manuscript.

## Conflict of Interest

The authors declare that the research was conducted in the absence of any commercial or financial relationships that could be construed as a potential conflict of interest.

## Abbreviations

AGC: Automatic gain control
AUC: area under the Receiver Operator Characteristic curve
BFI: biomarker of food intake
BMI: Body Mass Index
CRN: Clinical Research Network
EPIC: European Prospective Investigation into Cancer and Nutrition
FFQ: food frequency questionnaire
FIE-HRMS: flow infusion electrospray ionization—high resolution mass spectrometry
FMV: first morning void
HCD: Higher-energy collision dissociation
IPAQ: International Physical Activity Questionnaire
ISRCTN: International Standard Randomized Controlled Trial Number
MAIN: Metabolomics at Aberystwyth, Imperial and Newcastle
MDS: Multi-dimensional scaling
MRC: Medical Research Council
MS: mass spectrometry
MSI: Metabolomics Standards Initiative
MS^n^: Tandem mass spectrometry
NDNS: National Diet and Nutrition Survey
PA: physical activity
RF: Random Forest
ROC: Receiver Operator Characteristic
SD: Standard deviation
TMAO: Trimethylamine-N-oxide
UHPLC-HRMS: Ultra High Performance Liquid Chromatography-High Resolution MS.
