# Supplementary material for: Design and Characterisation of a Randomized Food Intervention That Mimics Exposure to a Typical UK Diet to Provide Urine Samples for Identification and Validation of Metabolite Biomarkers of Food Intake
Source: Front Nutr. 2020 Oct 21;7:561010. doi: 10.3389/fnut.2020.561010 (PMC7609501; doi:10.3389/fnut.2020.561010)
Supplement: Supplementary file 1 [file Table_1.DOCX]

**Supplementary** **Table S1.** Eligibility criteria for the MAIN Newcastle Study.

| Inclusion criteria | - Male and female - Age 18 - 80 years - BMI 18.5 - 29.9kg/m^2^ |
| --- | --- |
| Exclusion criteria | - Weight change of more than 3kg in the preceding 2 months - Current smoker - Substance abuse - Excess alcohol intake - Pregnancy - Diabetes mellitus - Cardiovascular disease (heart attack, heart disease or heart problems) - Current cancer - Gastrointestinal disease e.g. inflammatory bowel disease or irritable bowel syndrome - Kidney disease - Liver disease - Pancreatitis - Food allergy - Use of medications likely to interfere with energy metabolism, appetite regulation and hormonal balance, including but not exclusive to:   - anti-inflammatory drugs (NSAIDs)   - steroids or other immunosuppressive medication   - androgens   - phenytoin   - erythromycin   - thyroid hormones |
