# Supplementary material for: Design and Characterisation of a Randomized Food Intervention That Mimics Exposure to a Typical UK Diet to Provide Urine Samples for Identification and Validation of Metabolite Biomarkers of Food Intake
Source: Front Nutr. 2020 Oct 21;7:561010. doi: 10.3389/fnut.2020.561010 (PMC7609501; doi:10.3389/fnut.2020.561010)
Supplement: Supplementary file 2 [file Table_2.DOCX]

**Supplementary** **Table S2**. An example daily menu plan.

| Meal | Time | Menu |
| --- | --- | --- |
| Breakfast | 08:00 – 10:00 | Cup of whole bean instant coffee  Glass of white grape juice  Quality pork sausage, grilled  Whole meal bread roll with sunflower spread  Bowl of cornflakes with soya milk |
| Lunch | 12:00 – 14:00 | Cup of whole bean instant coffee  Cheese sandwich (one wholemeal bread roll with sunflower spread and spreadable cheese)  Sausage roll  Red pepper crudités  White grapes |
| Afternoon tea | 16:00 – 16:30 | Cup of whole bean instant coffee  White grapes  Strawberries |
| Dinner | 18:00 – 20:00 | 100% beef steak burger  Oven chips  Coleslaw  Natural yoghurt  Strawberries  Glass of sparkling white grape juice |

Three full-day menu plans, including a breakfast, lunch, afternoon snack and dinner, for each of two experimental periods were designed. Two 500ml bottles of water were also provided for each experimental day.
