# Supplementary material for: Design and Characterisation of a Randomized Food Intervention That Mimics Exposure to a Typical UK Diet to Provide Urine Samples for Identification and Validation of Metabolite Biomarkers of Food Intake
Source: Front Nutr. 2020 Oct 21;7:561010. doi: 10.3389/fnut.2020.561010 (PMC7609501; doi:10.3389/fnut.2020.561010)
Supplement: Supplementary file 3 [file Table_3.DOCX]

**Supplementary** **Table S3.** Summary of data and biological samples collected during Study 1.

| Data collected / measures | Study time point | | | | | | |
| --- | --- | --- | --- | --- | --- | --- | --- |
|  | Screening | Experimental week 1 | | | Experimental week 2 | | |
|  |  | Pre day visit | Between visits | Post day visit | Pre day visit | Between visits | Post day visit |
| Demographics (age, sex) & self-reported anthropometrics | X |  |  |  |  |  |  |
| Eligibility criteria (medical history, medications, supplements, diet & lifestyle) | X |  |  |  |  |  |  |
| Written consent |  | X |  |  |  |  |  |
| Randomisation |  | X |  |  |  |  |  |
| One day food diary |  | X |  |  | X |  |  |
| Height, weight & waist circumference |  | X |  | X | X |  | X |
| Food frequency questionnaire |  | X |  |  |  |  |  |
| IPAQ physical activity questionnaire |  | X |  |  | X |  |  |
| Dietary compliance record |  |  | X |  |  | X |  |
| Meal time record |  |  | X |  |  | X |  |
| Urine samples |  |  | X | X |  | X | X |
| Urine sample collection record |  |  | X | X |  | X | X |
| Blood sample (plasma, serum & whole blood) |  |  |  | X |  |  | X |

IPAQ, International Physical Activity Questionnaire
